# A Comparison of Disease Burden in Rheumatoid Arthritis, Psoriatic Arthritis and Axial Spondyloarthritis

**DOI:** 10.1371/journal.pone.0123582

**Published:** 2015-04-08

**Authors:** Brigitte Michelsen, Ragnhild Fiane, Andreas P. Diamantopoulos, Dag Magnar Soldal, Inger Johanne W. Hansen, Tuulikki Sokka, Arthur Kavanaugh, Glenn Haugeberg

**Affiliations:** 1 Department of Rheumatology, Hospital of Southern Norway Trust, Kristiansand, Norway; 2 Norwegian University of Science and Technology, Trondheim, Norway; 3 Jyväskylä Central Hospital, Jyväskylä, Finland; 4 Division of Rheumatology, Allergy, Immunology, University of California San Diego, San Diego, California, United States of America; 5 Hospital of Southern Norway Trust, Faculty of Health and Sport Sciences, University of Agder, Kristiansand, Norway; University of Texas Southwestern Medical Center, UNITED STATES

## Abstract

**Objective:**

The main objective of this study was to compare disease burden in rheumatoid arthritis (RA), psoriatic arthritis (PsA) and axial spondyloarthritis (ax-SpA).

**Methods:**

In this cross-sectional study, all the RA (1093), PsA (365) and ax-SpA (333) patients who visited the out-patient clinic of the Hospital of Southern Norway Trust during the year 2013 were included; the RA patients all had a RA diagnosis verified by the treating rheumatologist, the PsA patients all fulfilled the ClASsification for Psoriatic ARthritis (CASPAR) criteria and the ax-SpA patients all fulfilled the Assessment of SpondyloArthritis international Society (ASAS) classification criteria for ax-SpA. Patient-reported health status, demographic variables, medications, and composite scores of disease activity were assessed. The main analyses were performed using General Linear Models adjusted for age, sex and multiple comparisons. Correlation analyses were performed using Spearman’s rho.

**Results:**

The reported pain, joint pain, patient’s global assessment and fatigue were similar in PsA and ax-SpA, but significantly lower in RA. The 28-joint Disease Activity Score (DAS28) (0.3±0.1, p = 0.003), Clinical Disease Activity Index (CDAI) (1.0±0.4, p = 0.028) and Routine Assessment of Patient Index Data 3 (RAPID3) (0.4±0.1, p = 0.004) were all significantly higher in PsA vs. RA. RAPID3 showed moderate to high correlation with DAS28 (rho = 0.521, p<0.001) and CDAI (rho = 0.768, p<0.001) in RA and PsA, and with Bath Ankylosing Spondylitis Disease Activity Index (BASDAI) (rho = 0.902, p<0.001) and Bath Ankylosing Spondylitis Functional Index (BASFI) (0.865, p<0.001) in ax-SpA and PsA.

**Conclusion:**

In conclusion, patient- reported outcome measures were similar in our population of PsA and ax-SpA patients, but significantly lower for the RA patients. Composite disease activity measures were lower in RA than in PsA and ax-SpA, but the magnitude of these differences was small and probably not of clinical significance. Our study indicates that disease burden in RA, PsA and ax-SpA may be more similar than previously demonstrated.

## Introduction

Rheumatoid arthritis (RA), psoriatic arthritis (PsA) and axial spondyloarthritis (ax-SpA) are the most common inflammatory rheumatic diseases in ordinary outpatient clinics. In Norway the prevalence reported is 0.5% for RA [1], 0.2% for PsA [2] and 0.2% for ankylosing spondylitis (AS) [3]. These inflammatory disorders are characterised by different clinical, laboratory and imaging hallmarks. RA is characterised by symmetric and erosive arthritis typically affecting small and medium-sized joints [4]. PsA is a more heterogeneous inflammatory disease which may present as mild monoarthritis or severe polyarthritis and may also involve the axial skeleton and the entheses [5,6]. AS is a systemic inflammatory disorder that affects the sacroiliac joints and the spine, and can also affect peripheral joints and entheses [7]. Axial spondyloarthritis (ax-SpA) is a common term for inflammatory joint disorders with inflammation in axial skeleton and may include AS, undifferentiated spondyloarthritis and PsA with axial involvement [8,9]. In RA, bone involvement is characterized by erosions, whereas in PsA and ax-SpA bone involvement also includes signs of bone new formation [4,10,11].

Major clinical improvements have over the last years been achieved in the outcome of inflammatory rheumatic diseases. This has been attributed to various factors [7,12–15]. For example, better treatments are available and early aggressive treatment strategies have been more commonly adopted, including the treat to target (T2T) strategy for both RA and SpA. In addition, there have been changes in environmental exposures, e.g. smoking habits, which also may have altered outcome of inflammatory joint disorders.

Due to heterogeneity in expression of inflammatory rheumatic diseases, it is expected that there may be differences in disease burden between RA, PsA and ax-SpA. Few studies have compared clinical findings and patient- reported outcome measures in the three disease entities RA, PsA and ax-SpA, although several studies have compared health-related quality of life in these diseases [16–19].

Thus, the main objective of this study was to compare patient-reported health status and composite scores of disease activity in unselected patient populations of RA, PsA and ax-SpA, recruited from an ordinary out-patient clinic in Norway. Secondary objectives were to compare demographics and the use of Disease-Modifying AntiRheumatic Drugs (DMARDs) in RA, PsA and ax-SpA.

## Methods

### Study population

This is a cross-sectional study of consecutive RA, PsA and ax-SpA patients who visited the outpatient clinic of the Hospital of Southern Norway Trust during the year 2013. The RA, PsA and ax-SpA patients in the out-patient clinic were monitored by use of the GoTreatIT Rheuma computer software system (www.diagraphit.com) as part of the ordinary clinical care. The RA patients (1093) all had a RA diagnosis verified by the treating rheumatologist, but were not evaluated using the American College of Rheumatology (ACR) classification criteria on a regular basis [20]. 66.8% (682/1021) of the RA patients were rheumatoid factor (RF) positive, 69.4% (697/1004) anti-citrullinated peptide antibodies (ACPA) positive and 75.3% (747/992) positive to RF, ACPA or both. The PsA patients (365) all fulfilled the ClASsification for Psoriatic ARthritis (CASPAR) criteria [21]. PsA patients with axial inflammation were counted to the ax-SpA group. Patients with AS (266), PsA with axial inflammatory affection (22) or non-radiographic ax-SpA (45) were included in the ax-SpA group. They all (333) fulfilled the ASAS classification criteria for ax-SpA [8]. 85.6% (n = 250/292) of the ax-SpA and 27.1% (n = 45/166) of the PsA patients were HLA-B27 positive.

### Data collection

Patients completed computerized questionnaires assessing health status and demographic variables. The demographic data included age, gender, weight, height, body mass index (BMI), years of education, current smoking status and employment status.

Employment status was assessed and divided into 5 groups; Full-time employment, part-time employment (including part- time work/ disabled pensioner, part-time work/ sick leave, part-time work/ unemployed), disabled pensioner, not working for other reasons (including medical/ occupational rehabilitation, maternity/ paternity leave, student, sick leave and unemployed) and pensioner.

Patients’ global assessment, pain, joint pain, spine pain, spine pain at night and fatigue was reported on a visual analogue scale (VAS, 0–100 mm). Morning stiffness was reported in hours (0–6). Patients’ physical function was assessed by the Modified Health Assessment Questionnaire (MHAQ, 0–3) [22]. For calculation of the Routine Assessment of Patient Index Data 3 (RAPID3, 0–10) we used pain, patient’s global assessment and MHAQ, as MHAQ and not MDHAQ was assessed in the outpatient clinic [23]. Disease activity in ax-SpA and PsA was also evaluated by employing the Bath Ankylosing Spondylitis Disease Activity Index (BASDAI, 0–10) and the Bath Ankylosing Spondylitis Functional Index (BASFI, 0–10) [24,25].

C-reactive protein (CRP) (turbidimetry, lowest detectable value 1 mg/L) and erythrocyte sedimentation rate (ESR, mm/hr) (Westergren’s method) were measured. 28-swollen (28-SJC) and tender (28-TJC) joint count were performed by a rheumatologist or a specially trained nurse, who also assessed evaluator’s global assessment (VAS, 0–100 mm). The Clinical Disease Activity Index (CDAI) and 28-joint Disease Activity Score (DAS28-ESR) with (DAS28-ESR(4)) and without (DAS28-ESR(3)) patient’s global assessment was calculated by the computer system [26,27].

We retrieved data on current and previous use of conventional synthetic DMARDs (csDMARDs), biologic DMARDs (bDMARDs) and prednisolon/ prednisone.

### Statistics

Statistical analyses were performed using SPSS version 16.0 (SPSS Inc, Chicago, IL, USA).

We performed descriptive analyses of the crude estimates. The threshold for significance was set at p<0.05. Data for continuous demographics were analysed using One-way ANOVA with post-hoc tests (Tukey HSD when homogeneity of variance, Dunnett’s T3 when violation of homogeneity of variances). Data for categorical demographics and medication were analysed using Pearson Chi-Square test for independence. The main analyses were performed using a General Linear Model with adjustments for age and sex. The different patient-reported outcome measures, composite scores, ESR and CRP were used as the dependent variable in the General Linear Model. Sex and diagnoses were put as fixed factors and age as a covariate factor. Pairwise comparisons between the RA, PsA and ax-SpA groups were performed using Bonferroni adjustment for multiple comparisons. The unadjusted analyses were performed by use of One-way ANOVA with post-hoc tests (Tukey HSD when homogeneity of variance, Dunnett’s T3 when violation of homogeneity of variances) or independent t-test as appropriate. Correlation between RAPID3 and DAS28, CDAI, BASDAI and BASFI were investigated using Spearman’s rho.

### Ethics Board Approval

This study has been approved by the Norwegian Regional Committees for Medical and Health Research Ethics (Regional komité for medisinsk og helsefaglig forskningsetikk Midt-Norge 2010/3078) as a quality assurance study of established treatment with data obtained from the hospital medical record systems. The data were anonymized prior to analysis. Patient consent was not required according to Norwegian law, and confirmed by the Norwegian Regional Committees for Medical and Health Research Ethics.

## Results

### Study population

S1 Table displays the demographic characteristics of the study population of 1093 RA, 365 PsA and 333 ax-SpA patients. The ax-SpA patients (48±12.9 years) were significantly (p<0.001) younger than the PsA (55±12.4 years) and the RA patients (63±13.8 years).

Although the RA patients were older than the ax-SpA patients, the two groups did not differ in disease duration (RA: 12.4±10.6 years, ax-SpA: 13.0±11.8 years, p = 0.777). The PsA patients had significantly (p<0.001) shorter disease duration (9.9 ± 8.2) than the RA and the ax-SpA patients. The RA patients had significantly (p<0.001) less education (11.4±3.6 years) than the PsA (12.4±3.6 years) and the ax-SpA patients (12.8±3.5 years).

The RA patients were predominantly female (68.5%), the ax-SpA patients predominantly male (66.7%), whereas the gender distribution in the PsA group was equally balanced with 49.3% female. There was no statistically significant difference in smoking habits between the diagnosis groups.

In the RA group a higher percentage of the patients were pensioners (41.4%) compared to the PsA (16.7%) and the ax-SpA (7.4%) group. When considering only the working age group, there was still a highly significant difference in employment status between the RA, PsA and ax-SpA group; 26.0% of the RA patients, 32.0% of the PsA patients and 44.3% of the ax-SpA patients had full-time employment. The highest proportion of disabled pensioners was found in the RA group (38.7%).

The PsA patients had significantly higher BMI (27.6±4.3 kg/m2) than the RA (25.7±5.4 kg/m2, p<0.001) and the ax-SpA (26.4±4.3 kg/m2, p = 0.002) patients; also when adjusted for age and gender.

### Patient reported outcome measures and laboratory assessments

S2 Table shows patient-reported outcome measures and laboratory measures, both unadjusted values and values adjusted for sex and age. In the adjusted analysis patient’s mean global assessment was significantly (p<0.001) lower for RA patients (31.7±0.9 mm) than for PsA (39.2±1.4 mm) and ax-SpA (41.0±1.6 mm) patients, whereas no significant difference was found for evaluator’s global assessment between the diagnosis groups. RA patients also reported significantly less pain (31.2±0.9 mm) than the PsA (35.8±1.4 mm) and the ax-SpA (39.0±1.5 mm) patients (p = 0.015). Similar results were also seen for joint pain (RA: 29.6±0.8 mm, PsA: 35.6±1.3 mm, ax-SpA: 38.4 ± 1.5 mm). RA patients also experienced significantly less fatigue (34.3±1.0) than the PsA (44.3±1.6) and the ax-SpA (46.8±1.8) patients (p<0.001).

Further, the ax-SpA patients reported significantly more spine pain and spine pain at night than the RA and the PsA patients. In terms of patients’ global evaluation, pain, joint pain and fatigue, the PsA patients had similar values to the ax-SpA patients, while the RA patients had significantly lower values. These differences in patient reported outcome measures were still significant when adjusted for current use of bDMARDs, steroids and disease duration.

There was no significant difference in morning stiffness, ESR, CRP or MHAQ between the RA, PsA and ax-SpA groups. BASDAI (0.5 ± 0.2, p = 0.009) and BASFI (0.4 ± 0.1, p = 0.030) were significantly higher in the ax-SpA group than in the PsA group.

Supplementary subgroup analyses of patient-reported outcome measures and laboratory measures were performed for only seropositive RA patients compared to PsA and ax-SpA patients. This did not change the main outcomes (S3 Table).

### Composite scores

In the sex and age adjusted analyses, DAS28-ESR(4) was significantly (p = 0.003) lower for the RA patients (2.59±0.04) than for the PsA patients (2.85±0.07). The CDAI results were similar to the DAS28-ESR(4): 6.08±1.5 in the RA group, 7.03±0.4 in the PsA group, p = 0.028 (S4 Table). This despite the fact that PsA patients (0.51±0.1), were found to have significantly (p = 0.016) lower 28-SJC than the RA patients (0.79±0.1). There was no significant (0.42, p = 0.053) difference in 28-TJC between the PsA and RA patients. RAPID 3 was significantly lower in RA (2.5±0.1) than in PsA (2.9±0.1, p = 0.004) and ax-SpA patients (3.1±0.1, p<0.001). RAPID3 showed moderate correlation with DAS28-ESR(4) (rho = 0.521, p<0.001) and CDAI (rho = 0.768, p<0.001) for the RA and the PsA patients. RAPID3 was also strongly correlated to BASDAI (rho = 0.902, p<0.001) and BASFI (0.865, p<0.001) for the ax-SpA and PsA patients.

Subgroup analyses of CDAI, TJC28, SJC28, DAS28-ESR(3) and DAS28-ESR(4) were also performed, including only seropositive RA patients compared to PsA and ax-SpA patients. These subgroup analyses did not affect the main outcomes, except for DAS28-ESR(3), for which the mean difference between the RA and the PsA patients went from 0.17, p = 0.046 to 0.09, p = 0.312. For DAS28-ESR(4) the mean difference between the RA and the PsA patients went from 0.26, p = 0.003 to 0.20, p = 0.036 (S5 Table).

### Treatment

S6 Table shows current and previous use of bDMARDs and csDMARDs. The current use of bDMARDs was significantly more frequent (p = 0.001) in the ax-SpA (45.3%), than in the PsA (33.4%) and the RA group (34.5%). Former use of bDMARDs was also more frequent (p = 0.002) in the ax-SpA (53.8%) than in the PsA (41.4%) and the RA group (44.4%). The current use of TNF inhibitors was 45.0% in the ax-SpA, 31.8% in the PsA and 21.0% in the RA group. Previous and current use of csDMARDs was more frequent in the RA group compared to the PsA group (previous use: 92.1% versus 84.4%, p<0.001, current use: 61.1% versus 52.9%, p = 0.003).

## Discussion

The main finding in this study is that the PsA and the ax-SpA patients reported more pain and fatigue than the RA patients. Interestingly, the reported pain, joint pain, patient’s global assessment and fatigue were similar for the PsA and the ax-SpA patients, but significantly lower for the RA patients. Further, the composite disease activity measures DAS28, CDAI and RAPID3 were higher in PsA than in RA, but the magnitude of these differences was small and probably not of clinical significance.

Our study provided a unique opportunity to compare the disease burden in RA, PsA and ax-SpA. There are a limited number of studies comparing these disease entities, and only few of them compare disease burden [16–19]. Zink et al compared quality of life and treatment among patients with RA, PsA and AS and reported a comparable burden of illness [16]. Similar disease activity, disability and reduced quality of life in patients with PsA compared to RA are previously reported [28–30]. To our knowledge this is one of the first large-scale studies in Scandinavia comparing patient-reported outcome measures and disease activity measures of consecutive RA, PsA and ax-SpA patients from an out-patient clinic.

Measuring patient perception of health is considered as a standard approach, not only in controlled clinical trials and observational longitudinal studies, but also in clinical practice [31–33]. Interestingly, from the patient perspective, pain is the area of health in which patients have their highest priorities for improvement [31]. This highlights the importance of improving our efforts to reduce pain in patients with inflammatory joint disorders.

The comparison of health status of patients with different inflammatory rheumatic disease entities may be challenging because of the different age and sex distributions and the different disease durations of these disorders. As expected, in our study the RA patients were older and predominantly female, while the ax-SpA patients were younger and predominantly male. Further, the PsA patients had significantly shorter disease duration than the RA and the ax-SpA patients. Sex differences in pain scores in inflammatory arthritis, with higher pain levels in females, have previously been reported [34]. As shown in S2 and S4 Tables, the adjusting for age and sex in the analyses implied major differences in significance when comparing some of the outcome variables between the diagnosis groups. There were e.g. significant differences in pain, joint pain and RAPID3 scores between the RA, PsA and ax-SpA patients when analyses were adjusted for age and sex, but not in the unadjusted analyses. Adjustment for disease duration and current use of bDMARDs and steroids did not change the outcomes.

Considering only the working age group, there were significant (p<0.001) differences in employment status; 44.3% of the ax-SpA, 32.0% of the PsA and 26.0% of the RA patients had full-time employment. A higher percentage of the RA patients (38.7%), than the PsA (28.7%) and the ax-SpA (17.3%) patients were disabled pensioners. In comparison, 9.4% of the general population in Norway were disabled pensioners in 2013 [35].

This is interesting, as the RA patients demonstrated significantly lower global assessment, pain, spine pain, spine pain at night, joint pain and fatigue than the PsA and the ax-SpA patients, values adjusted for age and gender. Louie et al compared functional limitations in RA and AS patients with uncertain results [36]. A similar overall malignancy incidence between RA and PsA patients has been described [37]. In a recent study RA, but not PsA patients, were found to have elevated risk of mortality compared to the general population [38]. A recent systematic review by Jamnitski et al. concluded that the cardiovascular risks were comparable in RA and PsA [39]. PsA has also been reported to be associated with higher rates of obesity, diabetes and hypertrigylceridemia compared with RA [40]. The PsA patients in this study displayed significantly higher BMI than the RA or the ax-SpA patients. Similar differences in BMI between PsA and RA patients have also previously been reported [40–42].

When the composite score RAPID3 was evaluated, the RA patients were found to have lower disease activity than the PsA and the ax-SpA patients. In our clinical practice, patients complete MHAQ (8 questions, a-h) and not MDHAQ (10 questions, a-j; question i: walking two miles, question j: participate in sports). Using MHAQ as a surrogate marker for MDHAQ in the RAPID3 calculations may at most contribute to an error in the final RAPID3 score (0–10) of 0.7. This limitation was, however, equal for the RA, PsA and ax-SpA patients.

The RA patients were also found to have lower disease activity than the PsA patients upon evaluation of DAS28-ESR(4) and CDAI. Upon evaluation of DAS28-ESR(3) the difference between the RA and the PsA patients was smaller. This reflects the fact that DAS28-ESR(3) excludes patients’ global assessment of disease activity, a measure that in this study was significantly lower for the RA than for the PsA patients.

DAS28 and CDAI scores are formally validated for RA [26,27], but not for PsA. However, DAS28 has been used in randomised clinical trials for assessment of disease activity in PsA [43,44]. In a busy clinical setting, DAS28 and CDAI appear attractive for evaluation of disease activity also in PsA, and are on a regular basis assessed for the PsA patients in our clinic.

RAPID3 correlated significantly with DAS28-ESR(4) (rho = 0.521, p<0.001) and CDAI (rho = 0.768, p<0.001). MHAQ did not differ significantly between the RA, PsA or ax-SpA groups. Hence in our population, the differences demonstrated for the RAPID3 score in the RA, PsA and ax-SpA groups are probably based on documented lower scores for patient’s assessment of pain and patient’s global assessment of health in the RA group.

Interestingly, although developed for RA, and ostensibly more focused on peripheral joints, the RAPID3 has been suggested to correlate well with BASDAI in AS patients [45]. In this study RAPID3 was also found to be strongly correlated with BASDAI (rho = 0.902, p<0.001) and BASFI (0.865, p<0.001) for the ax-SpA and PsA patients.

Remarkably, a higher proportion of the ax-SpA (45.3%) than the RA (34.5%) and the PsA (33.4%) patients were currently using bDMARDs (p = 0.001). The same trend was seen for former use of bDMARDs (ax-SpA: 53.8%, PsA 41.4%, RA 44.4%, p = 0.002) [46]. Possible reasons for this may be the younger age of the ax-SpA patients and socio-economical considerations; sufficient disease control may contribute to maintain work ability. Further, the effect of csDMARDs in ax-SpA is controversial; csDMARDs are not routinely prescribed for patients with only axial disease as they have not shown to be efficacious [47–49]. Interestingly, the RA patients used significantly more steroids than the PsA and the ax-SpA patients. Adjustment for current use of bDMARDs and steroids in the main analyses did not change the outcomes.

The use of a large routine database of unselected patients seen in ordinary rheumatology outpatient clinic allowed a unique opportunity of direct comparison between RA, PsA and ax-SpA patients. One limitation in our study is, however, the varying amount of missing data, as reported in S1–S5 Tables. Another limitation of this study is that the 1093 RA patients with clinical RA diagnosis had not been systematically evaluated for the ACR classification criteria. This is a limitation in interpreting the results, as patients who do not meet the ACR criteria may have a milder disease and better quality of life. However, as many as 75.3% of our RA patients were positive to RF, ACPA or both. In comparison, a review article from 2010 reported 50–80% of RA patients to be positive for RF, ACPA or both [4]. Subgroup analyses of the seropositive RA patients compared to the PsA and the ax-SpA patients did not change the main outcomes, except for DAS28-ESR(3), where the mean difference between the RA and the PsA patients went from 0.17, p = 0.046 to 0.09, p = 0.312. For DAS28-ESR(4) the mean difference between the RA and the PsA patients went from 0.26, p = 0.003 to 0.20, p = 0.036. The subgroup analyses are listed in S3 and S5 Tables. A further limitation of the study is that the outcome measures of employment and subjective disease activity measures may be confounded by multiple factors, e.g. coexisting medical conditions or social factors not evaluated in this study.

The differences in patient-reported outcome measures and disease activity between the RA and PsA patients in our study may have different explanations. In recent years, studies have shown differences in inflammatory processes involved in RA, PsA and ax-SpA [50]. Pain mechanisms in RA and PsA may be different, as the inflammatory process in PsA frequently involves entheses and spine [5]. Unfortunately, we did not have measures for entheses involvement, e.g. the MASES score, performed as routine care. Further, due to the rather frequent involvement of the DIP joints in PsA, the use of 28 joint count instead of e.g. the 66/68 joint count may have underreported clinical inflammatory joint involvement in PsA.

In conclusion, patient reported outcome measures were similar in our population of PsA and ax-SpA patients, but significantly lower for the RA patients. Composite disease activity measures were lower in RA than in PsA and ax-SpA, but the magnitude of these differences was small and probably not of clinical significance. Our study indicates that disease burden in RA, PsA and ax-SpA may be more similar than previously demonstrated.

## Supporting Information

S1 Table Patient demographics and employment status in rheumatoid arthritis (RA), psoriatic arthritis (PsA) and axial spondyloarthritis (ax-SpA). Data for continuous variables are shown as mean ± SD. a: RA- PsA, b: RA- axSpA, c: PsA- ax-SpA. *One-way ANOVA with post-hoc tests (Tukey HSD when homogeneity of variance, Dunnett’s T3 when violation of homogeneity of variances). ** Pearson Chi-Square tests.
(DOCX)

S2 Table Patient-reported outcome measures and laboratory inflammatory markers in rheumatoid arthritis (RA), psoriatic arthritis (PsA) and axial spondyloarthritis (ax-SpA). a: RA- PsA, b: RA- ax-SpA, c: PsA- ax-SpA. Data are shown as mean ± SE. *One-way ANOVA with post-hoc tests (Tukey HSD when homogeneity of variance, Dunnett’s T3 when violation of homogeneity of variances) or independent t-test as appropriate. **General Linear Model, adjusted for age, sex and multiple comparisons (Bonferroni).
(DOCX)

S3 Table Subgroup analyses of patient-reported outcome measures and laboratory inflammatory markers in seropositive rheumatoid arthritis (RA), psoriatic arthritis (PsA) and axial spondyloarthritis (ax-SpA). a: Seropositive RA- PsA, b: Seropositive RA- ax-SpA, c: PsA- ax-SpA. Data are shown as mean ± SE. *One-way ANOVA with post-hoc tests (Tukey HSD when homogeneity of variance, Dunnett’s T3 when violation of homogeneity of variances) or independent t-test as appropriate. **General Linear Model, adjusted for age, sex and multiple comparisons (Bonferroni).
(DOCX)

S4 Table Disease activity in rheumatoid arthritis (RA) and psoriatic arthritis (PsA). Data are shown as mean ± SE. * Independent t-test. ** General Linear Model, adjusted for age and sex.
(DOCX)

S5 Table Subgroup analyses of disease activity in seropositive rheumatoid arthritis (RA) and psoriatic arthritis (PsA). Data are shown as mean ± SE. * Independent t-test. ** General Linear Model, adjusted for age and sex.
(DOCX)

S6 Table Current and former use of biological and conventional synthetic DMARDSs in rheumatoid arthritis (RA), psoriatic arthritis (PsA) and axial spondyloarthritis (ax-SpA). Pearson Chi-Square tests for independence.
(DOCX)
