# Supplementary material for: A Comparison of Disease Burden in Rheumatoid Arthritis, Psoriatic Arthritis and Axial Spondyloarthritis
Source: PLoS One. 2015 Apr 8;10(4):e0123582. doi: 10.1371/journal.pone.0123582 (PMC4390320; doi:10.1371/journal.pone.0123582)
Supplement: S1 Table — Data for continuous variables are shown as mean ± SD. a: RA- PsA, b: RA- axSpA, c: PsA- ax-SpA. *One-way ANOVA with post-hoc tests (Tukey HSD when homogeneity of variance, Dunnett’s T3 when violation of homogeneity of variances). ** Pearson Chi-Square tests. (DOCX) [file pone.0123582.s001.docx]

**S1 Table.** Patient demographics and employment status in rheumatoid arthritis (RA), psoriatic arthritis (PsA) and axial spondyloarthritis (ax-SpA).

| *Demographics* | | **RA**  **(n=1093)** | **PsA (n=365)** | **ax-SpA (n=333)** | **p** | |
| --- | --- | --- | --- | --- | --- | --- |
| **Age (years)** | | 63.0 ± 13.8 | 55.5 ± 12.4 | 48.1 ± 12.9 | <0.001^a,b,c^ * | |
| **Female (%)** | | 68.5 | 49.3 | 33.3 | <0.001 ** | |
| **Disease duration (years)** | | 12.4 ± 10.6 | 9.9 ± 8.2 | 13.0 ± 11.8 | <0.001^a,c^ *  0.777^b^ | |
| **Education (years)** | | 11.4 ± 3.6  (n=1077) | 12.4 ± 3.6  (n=358) | 12.8 ± 3.5  (n=323) | <0.001^a,b^ *  0.210^c^ | |
| **Current smoking (%)** | | 20.5 (n=222/1083) | 18.4  (n=66/359) | 23.1 (n=75/324) | 0.305 ** | |
| **Body mass index (kg/m^2^)** | | 25.7 ± 4.5  (n=1045) | 27.6 ± 4.3  (n=351) | 26.4 ± 4.3  (n=314) | <0.001^a^ *  0.259^b^  0.002^c^ | |
|  | |  |  |  |  | |
| *Employment status* | | **RA**  **(n=1084)** | **PsA (n=360)** | **ax-SpA (n=324)** | **p** | |
| **Full-time employment (%)** | | 15.2 | 26.7 | 41.0 | <0.001** | |
| **Part-time employment (%)** | | 13.3 | 21.4 | 22.5 |  |  |
| **Disabled pensioner (%)** | | 22.7 | 23.9 | 16.0 |  |  |
| **Not working,**  **other reasons (%)** | | 7.4 | 11.4 | 13.0 |  |  |
| **Pensioner (%)** | | 41.4 | 16.7 | 7.4 |  |  |
|  | | | | | | |
| *Employment status, excluding pensioners* | **RA**  **(n=635)** | | **PsA (n=300)** | **ax-SpA (n=300)** | | **p** |
| **Full-time employment (%)** | 26.0 | | 32.0 | 44.3 | | <0.001** |
| **Part-time employment (%)** | 22.7 | | 25.7 | 24.3 | |  |
| **Disabled pensioner (%)** | 38.7 | | 28.7 | 17.3 | |  |
| **Not working,**  **other reasons (%)** | 12.6 | | 13.7 | 14.0 | |  |

Data for continuous variables are shown as mean ± SD

a: RA- PsA, b: RA- axSpA, c: PsA- ax-SpA

*One-way ANOVA with post-hoc tests (Tukey HSD when homogeneity of variance, Dunnett’s T3 when violation of homogeneity of variances)

** Pearson Chi-Square tests
