# Supplementary material for: A Comparison of Disease Burden in Rheumatoid Arthritis, Psoriatic Arthritis and Axial Spondyloarthritis
Source: PLoS One. 2015 Apr 8;10(4):e0123582. doi: 10.1371/journal.pone.0123582 (PMC4390320; doi:10.1371/journal.pone.0123582)
Supplement: S2 Table — a: RA- PsA, b: RA- ax-SpA, c: PsA- ax-SpA. Data are shown as mean ± SE. *One-way ANOVA with post-hoc tests (Tukey HSD when homogeneity of variance, Dunnett’s T3 when violation of homogeneity of variances) or independent t-test as appropriate. **General Linear Model, adjusted for age, sex and multiple comparisons (Bonferroni). (DOCX) [file pone.0123582.s002.docx]

**S2 Table.** Patient-reported outcome measures and laboratory inflammatory markers in rheumatoid arthritis (RA), psoriatic arthritis (PsA) and axial spondyloarthritis (ax-SpA).

| **Patient-reported outcome measures and laboratory inflammatory**  **markers** | **Unadjusted values** | | | | **Sex and age adjusted values** | | | |
| --- | --- | --- | --- | --- | --- | --- | --- | --- |
|  | **RA**  **(n=1093)** | **PsA**  **(n=365)** | **ax-SpA**  **(n=333)** | **P*** | **RA**  **(n=1093)** | **PsA (n=365)** | **ax-SpA (n=333)** | **P**** |
| **Patient’s global assessment (mm)** | 34.1 ± 0.8  (n=1008) | 38.1 ± 1.4  (n=342) | 37.1± 1.5  (n=309) | 0.037^a^  0.176^b^  0.877^c^ | 31.7 ± 0.9  (n=1008) | 39.2 ± 1.4  (n=342) | 41.0 ± 1.6  (n=309) | <0.001^a,b^  1.000 ^c^ |
| **Evaluator’s global assessment (mm)** | 9.8 ± 0.4  (n=909) | 10.5 ± 0.6  (n=304) | 10.4 ± 0.9  (n=171) | 0.566 | 9.6 ± 0.4  (n=909) | 10.6 ± 0.7  (n=304) | 10.6 ± 0.9  (n=171) | 0.643 ^a^  1.000 ^b,c^ |
| **Pain (mm)** | 33.7 ± 0.8  (n=995) | 34.7 ± 1.3  (n=341) | 35.1 ± 1.4  (n=308) | 0.627 | 31.2 ± 0.9  (n=995) | 35.8 ± 1.4  (n=341) | 39.0 ± 1.5  (n=308) | 0.015^a^  <0.001^b^  0.334^c^ |
| **Joint pain (mm)** | 32.0 ± 0.8  (n=997) | 34.5 ± 1.3  (n=343) | 34.4 ± 1.3  (n=306) | 0.144 | 29.6 ± 0.8  (n=997) | 35.6 ± 1.3  (n=343) | 38.4 ± 1.5  (n=306) | 0.001 ^a^  <0.001^b^  0.457 ^c^ |
| **Spine pain (mm)** | 31.2 ± 1.0  (n=894) | 32.6 ± 1.5  (n=340) | 36.8 ± 1.5  (n=308) | 0.790^a^  0.004^b^  0.133^c^ | 28.2 ± 1.0  (n=894) | 33.8 ± 1.5  (n=340) | 41.2 ± 1.7  (n=308) | 0.007^a^  <0.001^b^  0.003^c^ |
| **Spine pain at night (mm)** | 22.0 ± 0.9  (n=896) | 26.2 ± 0.9  (n=342) | 33.0 ± 1.5  (n=308) | 0.037^a^  <0.001^b^  0.003^c^ | 20.4 ± 0.96  (n=896) | 26.6 ± 1.4 (n=342) | 34.8 ± 1.6  (n=308) | 0.001^a^  <0.001^b,c^ |
| **Morning stiffness (mm)** | 0.92 ± 0.04  (n=990) | 0.90 ± 0.07  (n=343) | 0.93 ± 0.07  (n=306) | 0.954 | 0.87 ± 0.04  (n=990) | 0.92 ± 0.07  (n=343) | 1.00 ± 0.08  (n=306) | 1.000^a,b^  0.41^c^ |
| **Fatigue (mm)** | 36.9 ± 1.0 (n=998) | 43.6 ± 1.7  (n=342) | 43.6 ± 1.8  (n=308) | 0.001^a^  0.002^b^  1.000^c^ | 34.3 ± 1.0  (n=998) | 44.3 ± 1.6  (n=342) | 46.8 ± 1.8  (n=308) | <0.001^a,b^  0.891^c^ |
| **MHAQ (0-3)** | 0.49 ± 0.02  (n=1004) | 0.43 ± 0.02  (n=343) | 0.41 ± 0.02  (n=308) | 0.081^a^  0.022^b^  0.940^c^ | 0.44 ± 0.02  (n=1004) | 0.45 ± 0.03  (n=343) | 0.49 ± 0.03  (n=308) | 1.000^a^  0.464^b^  0.823^c^ |
| **RAPID3 (0-10)** | 2.7 ± 0.1  (n=982) | 2.8 ± 0.1  (n=336) | 2.8 ± 0.1  (n=306) | 0.567 | 2.5 ± 0.1  (n=982) | 2.9 ± 0.1  (n=336) | 3.1 ± 0.1  (n=306) | 0.004^a^  <0.001^b^  0.674^c^ |
| **BASDAI (0-10)** | - | 3.2 ± 0.1  (n=330) | 3.5 ± 0.1  (n=302) | 0.216 | - | 3.2 ± 0.1  (n=330) | 3.6 ± 0.1  (n=302) | 0.009^c^ |
| **BASFI (0-10)** | - | 2.8 ± 0.1  (n=330) | 2.9 ± 0.1  (n=302) | 0.878 | - | 2.7 ± 0.1  (n=330) | 3.1 ± 0.2  (n=302) | 0.030^c^ |
| **ESR (mm/hr)** | 17.2 ± 0.5  (n=797) | 14.6 ± 1.0  (n=229) | 12.4 ± 1.0  (n=186) | 0.037^a^  <.001^b^  0.243^c^ | 16.2 ± 0.5  (n=797) | 15.2 ± 0.9  (n=229) | 14.7 ± 1.1  (n=186) | 1.000^a,c^  0.66^b^ |
| **CRP (mg/L)** | 6.3 ± 0.4  (n=832) | 4.5 ± 0.6 (n=241) | 5.4 ± 0.6  (n=188) | 0.040^a^  0.572^b^  0.581^c^ | 6.3 ± 0.4  (n=832) | 4.6 ± 0.7  (n=241) | 5.7 ± 0.8)  (n=188) | 0.083^a^  1.000^b^  0.733^c^ |

a: RA- PsA, b: RA- ax-SpA, c: PsA- ax-SpA. Data are shown as mean ± SE.

*One-way ANOVA with post-hoc tests (Tukey HSD when homogeneity of variance, Dunnett’s T3 when violation of homogeneity of variances) or independent t-test as appropriate

**General Linear Model, adjusted for age, sex and multiple comparisons (Bonferroni)
