# Supplementary material for: A Comparison of Disease Burden in Rheumatoid Arthritis, Psoriatic Arthritis and Axial Spondyloarthritis
Source: PLoS One. 2015 Apr 8;10(4):e0123582. doi: 10.1371/journal.pone.0123582 (PMC4390320; doi:10.1371/journal.pone.0123582)
Supplement: S4 Table — Data are shown as mean ± SE. * Independent t-test. ** General Linear Model, adjusted for age and sex. (DOCX) [file pone.0123582.s004.docx]

**S4 Table. Disease activity in rheumatoid arthritis (RA) and psoriatic arthritis (PsA).**

|  | **Unadjusted values** | | | **Sex and age adjusted values** | | |
| --- | --- | --- | --- | --- | --- | --- |
|  | **RA**  **(n=1093)** | **PsA**  **(n=365)** | **P*** | **RA**  **(n=1093)** | **PsA (n=365)** | **P**** |
| **CDAI** | 6.36 ± 0.21  (n=839) | 6.89 ± 0.35  (n=283) | 0.203 | 6.08 ± 0.22  (n=839) | 7.03 ± 0.37  (n=283) | 0.028 |
| **Tender joint**  **count (0-28)** | 1.39 ± 0.10  (n=915) | 1.86 ± 0.21  (n=305) | 0.043 | 1.35 ± 0.11  (n=915) | 1.77 ± 0.19  (n=305) | 0.053 |
| **Swollen joint**  **count (0-28)** | 0.84 ± 0.06  (n=915) | 0.49 ± 0.07  (n=305) | <0.001 | 0.79 ± 0.06  (n=915) | 0.51 ± 0.10  (n=305) | 0.016 |
| **DAS28-ESR(4)** | 2.69 ± 0.04  (n=706) | 2.77 ± 0.08  (n=207) | 0.307 | 2.59 (0.04)  (n=706) | 2.85 (0.08)  (n=207) | 0.003 |
| **DAS28-ESR(3)** | 2.60 ± 0.04  (n=730) | 2.63 ± 0.07  (n=213) | 0.669 | 2.52 ± 0.04  (n=730) | 2.69 ± 0.07  (n=213) | 0.046 |

Data are shown as mean ± SE

* Independent t-test

** General Linear Model, adjusted for age and sex
