# Supplementary material for: A Comparison of Disease Burden in Rheumatoid Arthritis, Psoriatic Arthritis and Axial Spondyloarthritis
Source: PLoS One. 2015 Apr 8;10(4):e0123582. doi: 10.1371/journal.pone.0123582 (PMC4390320; doi:10.1371/journal.pone.0123582)
Supplement: S5 Table — Data are shown as mean ± SE. * Independent t-test. ** General Linear Model, adjusted for age and sex. (DOCX) [file pone.0123582.s005.docx]

**S5 Table.** Subgroup analyses of disease activity in seropositive rheumatoid arthritis (RA)

and psoriatic arthritis (PsA).

|  | **Unadjusted values** | | | **Sex and age adjusted values** | | |
| --- | --- | --- | --- | --- | --- | --- |
|  | **Seropositive RA**  **(n=778)** | **PsA**  **(n=365)** | **P*** | **Seropositive RA**  **(n=778)** | **PsA (n=365)** | **P**** |
| **CDAI** | 6.35 ± 0.26  (n=601) | 6.89 ± 0.35  (n=283) | 0.221 | 6.07 ± 0.26  (n=601) | 7.00 ± 0.37  (n=283) | 0.045 |
| **Tender joint**  **count (0-28)** | 1.47 ± 0.12  (n=650) | 1.86 ± 0.21  (n=305) | 0.091 | 1.43 ± 0.14  (n=650) | 1.76 ± 0.19  (n=305) | 0.161 |
| **Swollen joint**  **count (0-28)** | 0.87 ± 0.07  (n=650) | 0.49 ± 0.07  (n=305) | <0.001 | 0.81 ± 0.07  (n=650) | 0.50 ± 0.10  (n=305) | 0.008 |
| **DAS28-ESR(4)** | 2.74 ± 0.05  (n=512) | 2.77 ± 0.08  (n=207) | 0.723 | 2.65 (0.05)  (n=512) | 2.85 (0.08)  (n=207) | 0.036 |
| **DAS28-ESR(3)** | 2.66 ± 0.05  (n=527) | 2.63 ± 0.07  (n=213) | 0.739 | 2.59 ± 0.05  (n=527) | 2.68 ± 0.07  (n=213) | 0.312 |

Data are shown as mean ± SE

* Independent t-test

** General Linear Model, adjusted for age and sex
