# Supplementary material for: A Comparison of Disease Burden in Rheumatoid Arthritis, Psoriatic Arthritis and Axial Spondyloarthritis
Source: PLoS One. 2015 Apr 8;10(4):e0123582. doi: 10.1371/journal.pone.0123582 (PMC4390320; doi:10.1371/journal.pone.0123582)
Supplement: S6 Table — Pearson Chi-Square tests for independence. (DOCX) [file pone.0123582.s006.docx]

**S6 Table.** Current and former use of biological and conventional synthetic DMARDSs in rheumatoid arthritis (RA), psoriatic arthritis (PsA) and axial spondyloarthritis (ax-SpA).

|  |  | **RA**  **(n=1093)** | **PsA (n=365)** | **Ax-SpA**  **(n=333)** | **p** |
| --- | --- | --- | --- | --- | --- |
| **Current use of** | **Biological DMARDs (%)** | 34.5 | 33.4 | 45.3 | 0.001 |
|  | **TNF-α inhibitors (%)** | 21.0 | 31.8 | 45.0 | <0.001 |
|  | **Conventional synthetic DMARDs (%)** | 61.1 | 52.9 | 6.9 | <0.001 |
|  | **Methotrexate (%)** | 51.0 | 37.0 | 3.9 | <0.001 |
|  | **Prednisolon or prednisone (%)** | 54.3 | 13.7 | 3.9 | <0.001 |
| **Former use of** | **Biological DMARDs (%)** | 44.4 | 41.4 | 53.8 | 0.002 |
|  | **TNF-α inhibitors (%)** | 41.2 | 40.0 | 53.8 | <0.001 |
|  | **Conventional synthetic DMARDs (%)** | 92.1 | 84.4 | 19.8 | <0.001 |
|  | **Methotrexate (%)** | 86.1 | 75.3 | 13.5 | <0.001 |
|  | **Prednisolon or prednisone (%)** | 80.7 | 30.4 | 11.1 | <0.001 |

Pearson Chi-Square tests for independence.
